# Supplementary figures and images for: Pre‐oviposition development of the brown anole (Anolis sagrei)
Source: Dev Dyn. 2025 Apr 17;255(2):145–67. doi: 10.1002/dvdy.70027 (PMC12873869; doi:10.1002/dvdy.70027)

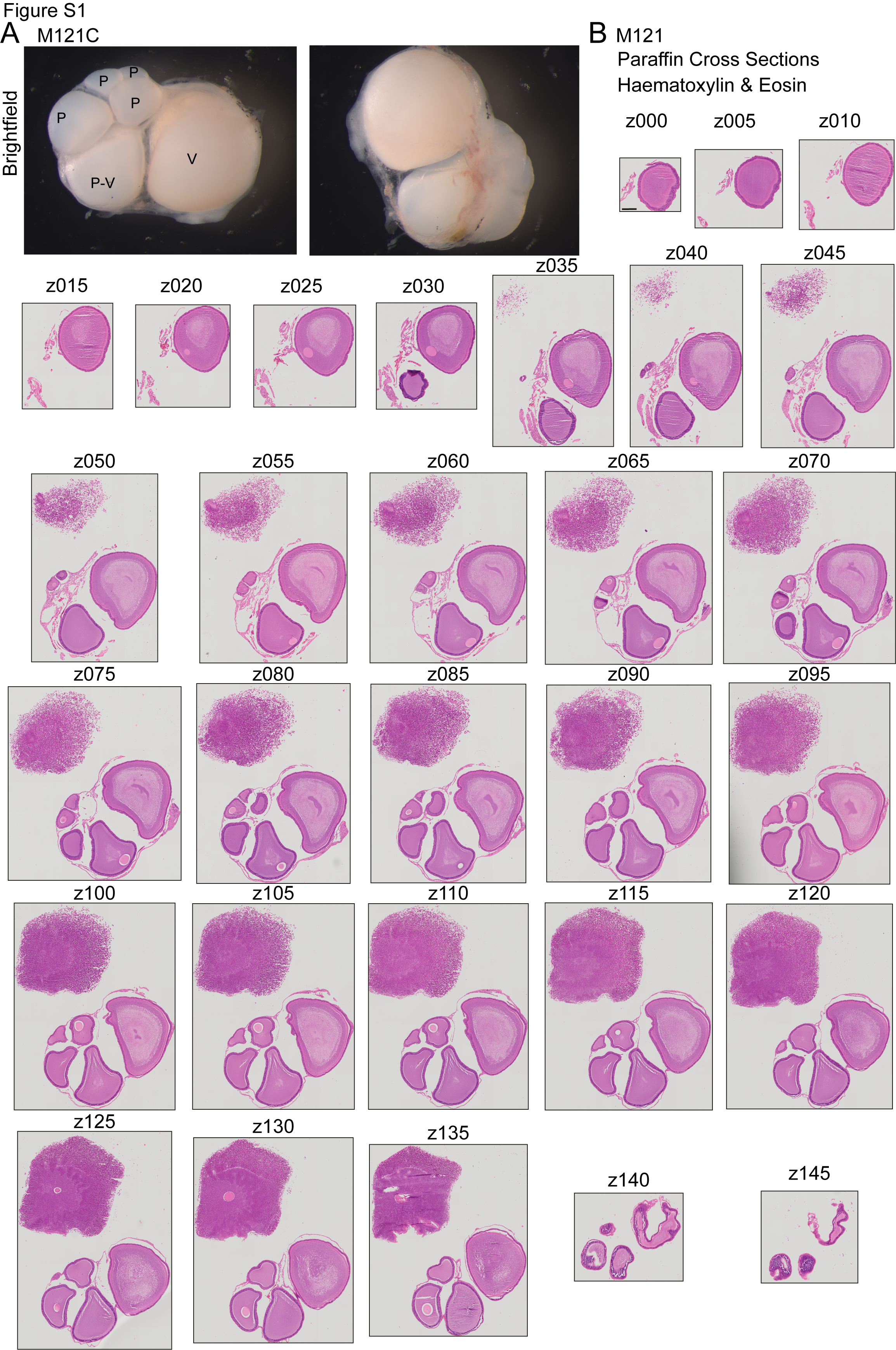

Supplement: Supplementary file 1 — FIGURE S1: Morphology of maturing follicles. (A) Brightfield images of follicles M121C. No scale bar. (B) Hematoxylin and eosin staining of paraffin cross sections of follicles M121C. Cross section number annotated in figure (z000–z145). Scale bar: 250 μm. All images have the same scale. [file DVDY-255-145-s014.tif]

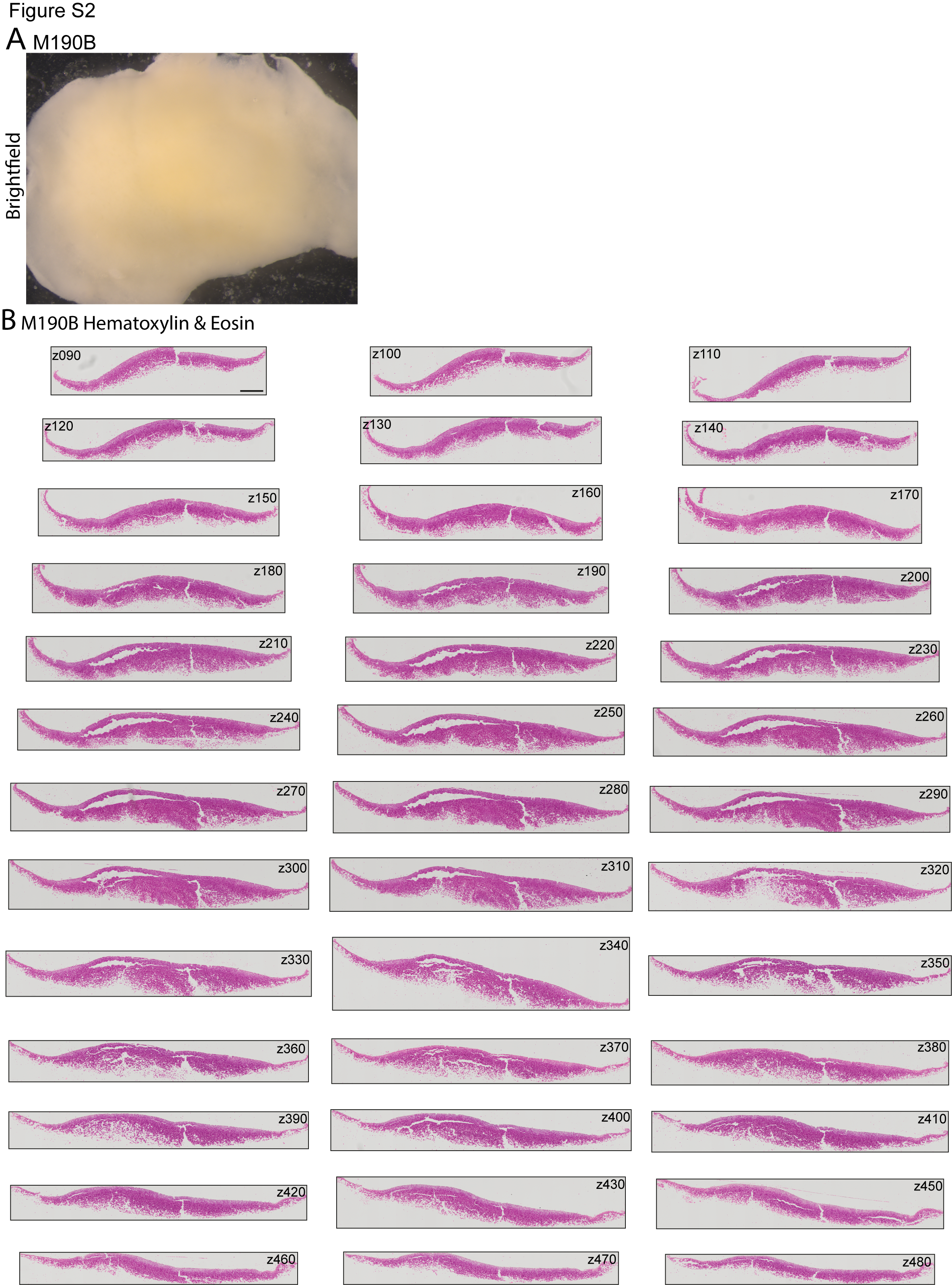

Supplement: Supplementary file 2 — FIGURE S2: Cross sections of cleavage stage M190B. (A) Brightfield image of M190B dorsal view. No scale bar. (B) Hematoxylin and eosin staining of paraffin cross sections of M190B. Cross section number annotated in figure (z090–z480). Scale bar: 400 μm. All images have the same scale. [file DVDY-255-145-s009.tif]

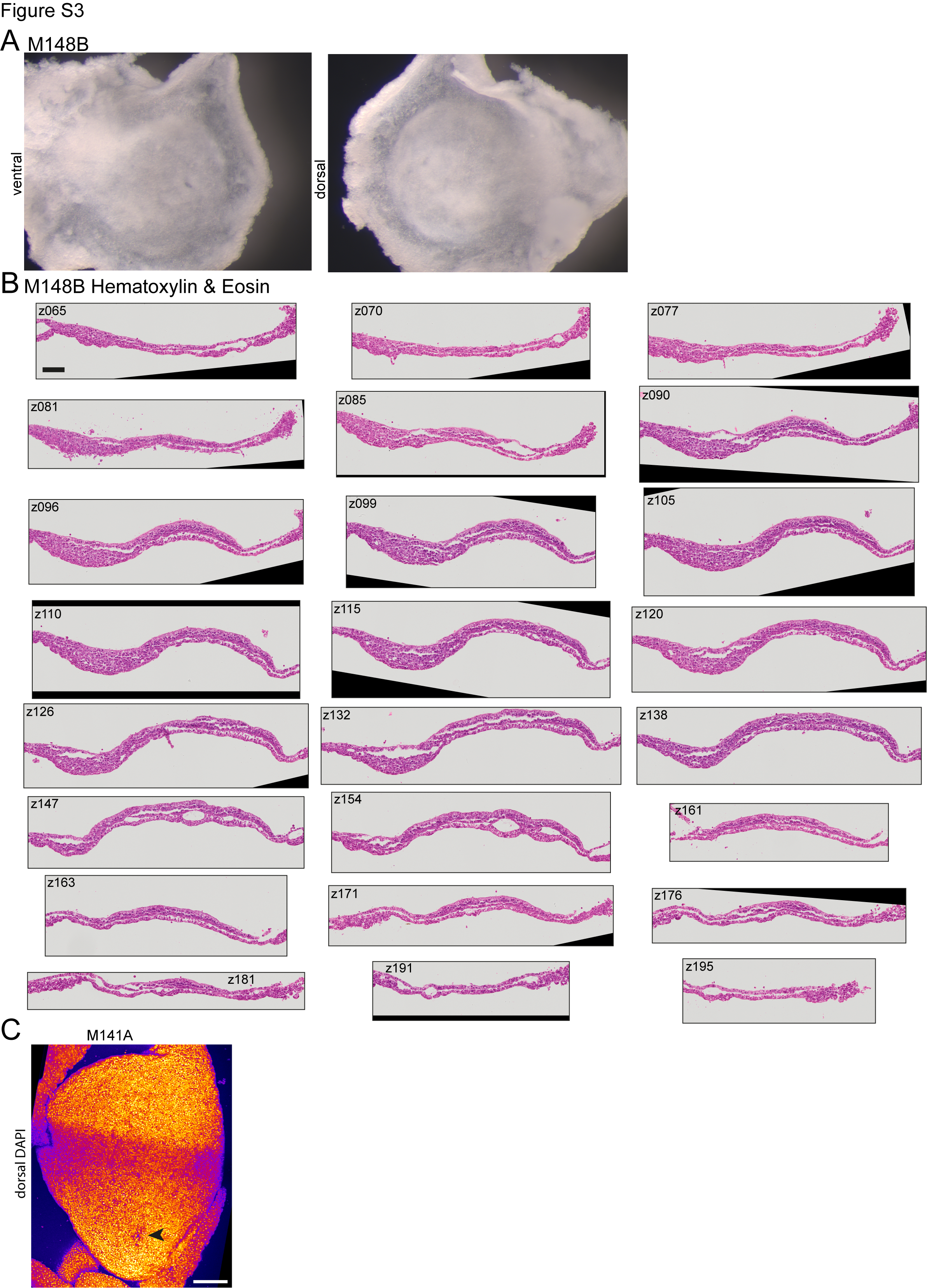

Supplement: Supplementary file 3 — FIGURE S3: Cross sections of gastrulation stage embryo M148B. (A) Brightfield images of M148B ventral and dorsal views. No scale bar. (B) Hematoxylin and eosin staining of paraffin cross sections of M148B. Cross section number annotated in figure (z065–z195). Scale bar: 100 μm. All images have the same scale. (C) Maximum intensity projection DAPI staining of M141A. Blastopore is indicated with arrow. Scale bar: 250 μm. [file DVDY-255-145-s004.tif]

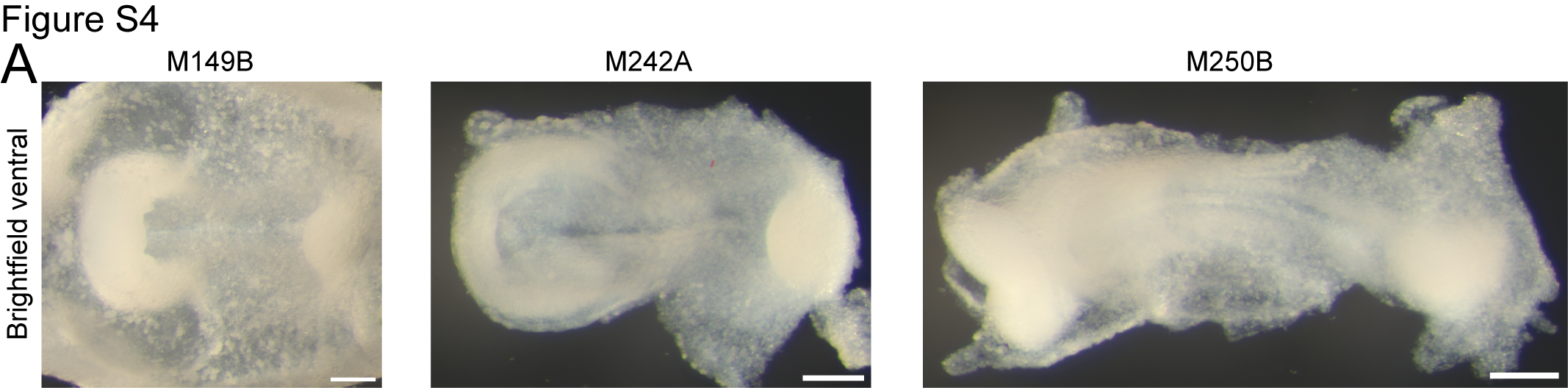

Supplement: Supplementary file 4 — FIGURE S4: Brightfield images of initiation of neurulation ventral views. (A) Brightfield images of ventral view of M149B, M242A, and M250B. Scale bar: 200 μm. [file DVDY-255-145-s002.tif]

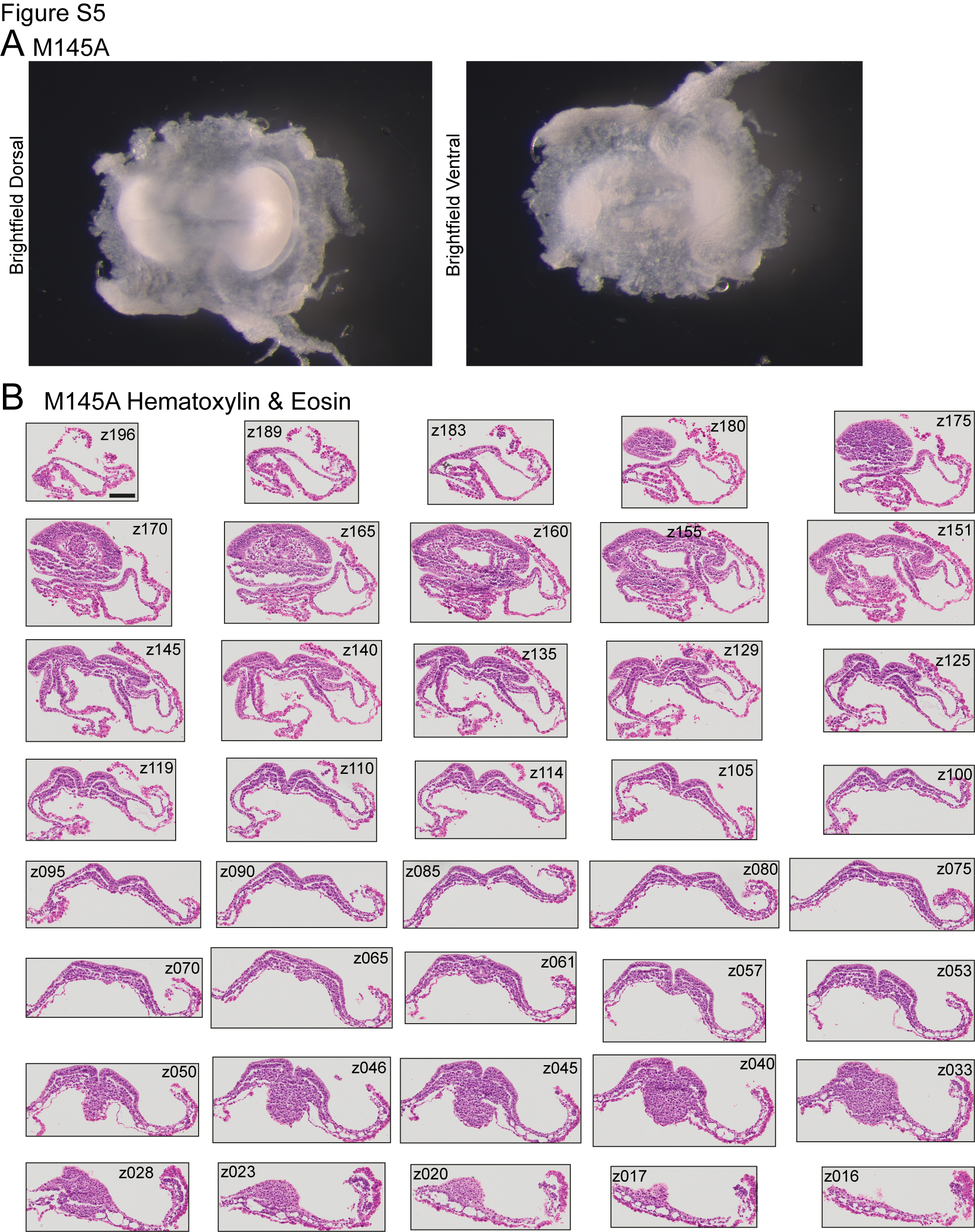

Supplement: Supplementary file 5 — FIGURE S5: Cross section of initiation of neurulation M145A. (A) Brightfield images of dorsal and ventral view of M145A. No scale bar. (B) Hematoxylin and eosin staining of paraffin cross sections of M145A. Cross section number annotated in figure (z196–z016 anterior to posterior). Scale bar: 100 μm. All images have the same scale. [file DVDY-255-145-s007.tif]

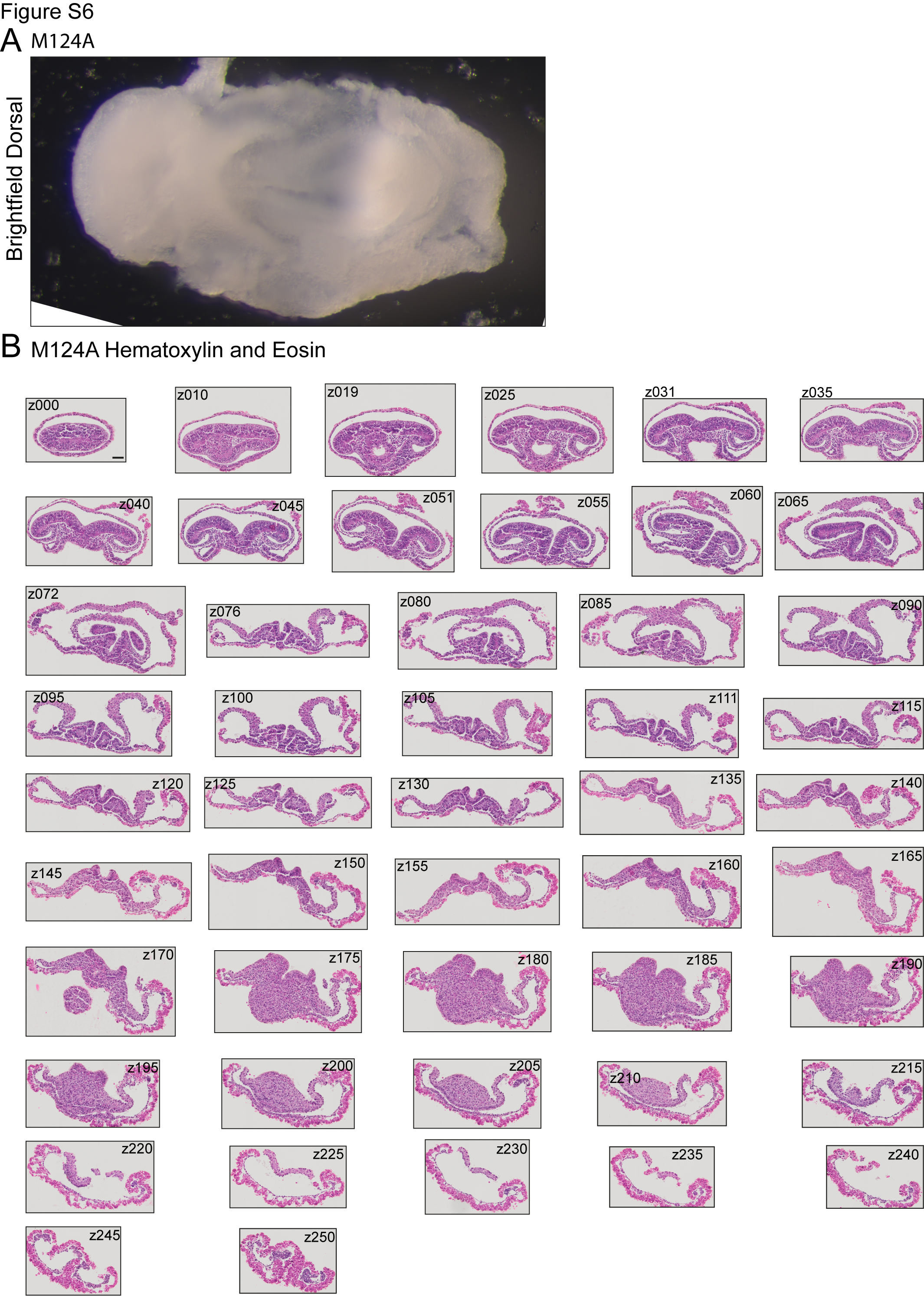

Supplement: Supplementary file 6 — FIGURE S6: Cross sections of early neurulation embryo M124A. (A) Brightfield image of dorsal view of M124A. No scale bar. (B) Hematoxylin and eosin staining of paraffin cross sections of M124A. Cross section number annotated in figure (z000–z250). Scale bar: 100 μm. All images have the same scale bar. [file DVDY-255-145-s015.tif]

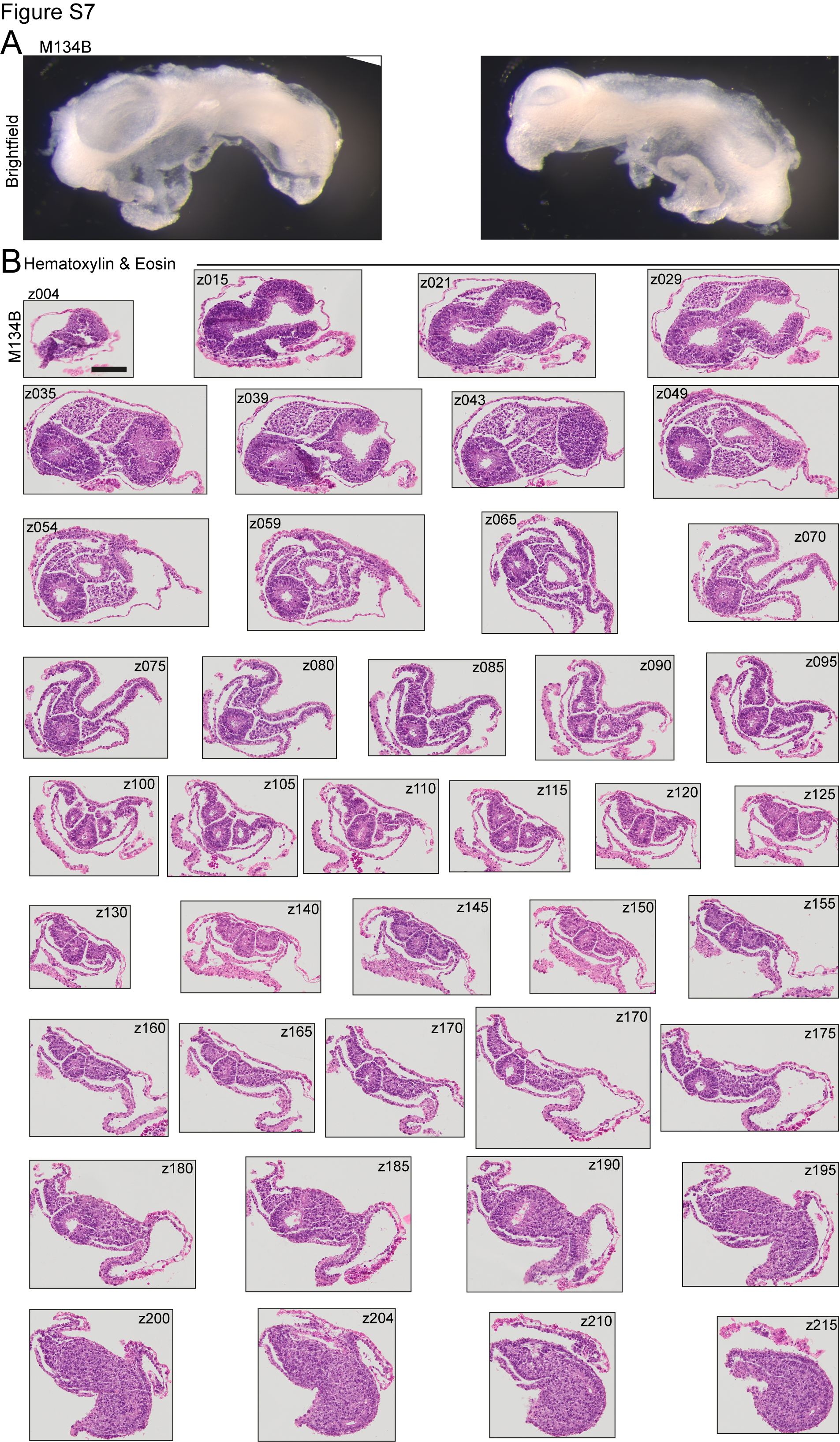

Supplement: Supplementary file 7 — FIGURE S7: Cross section of neurulation embryo M134B. (A) Brightfield image of right and left side of M134B. No scale bar. (B) Hematoxylin and eosin staining of paraffin cross sections of M134B. Cross section number annotated in figure (z004–z215). Scale bar: 100 μm. All images have the same scale bar. [file DVDY-255-145-s008.tif]

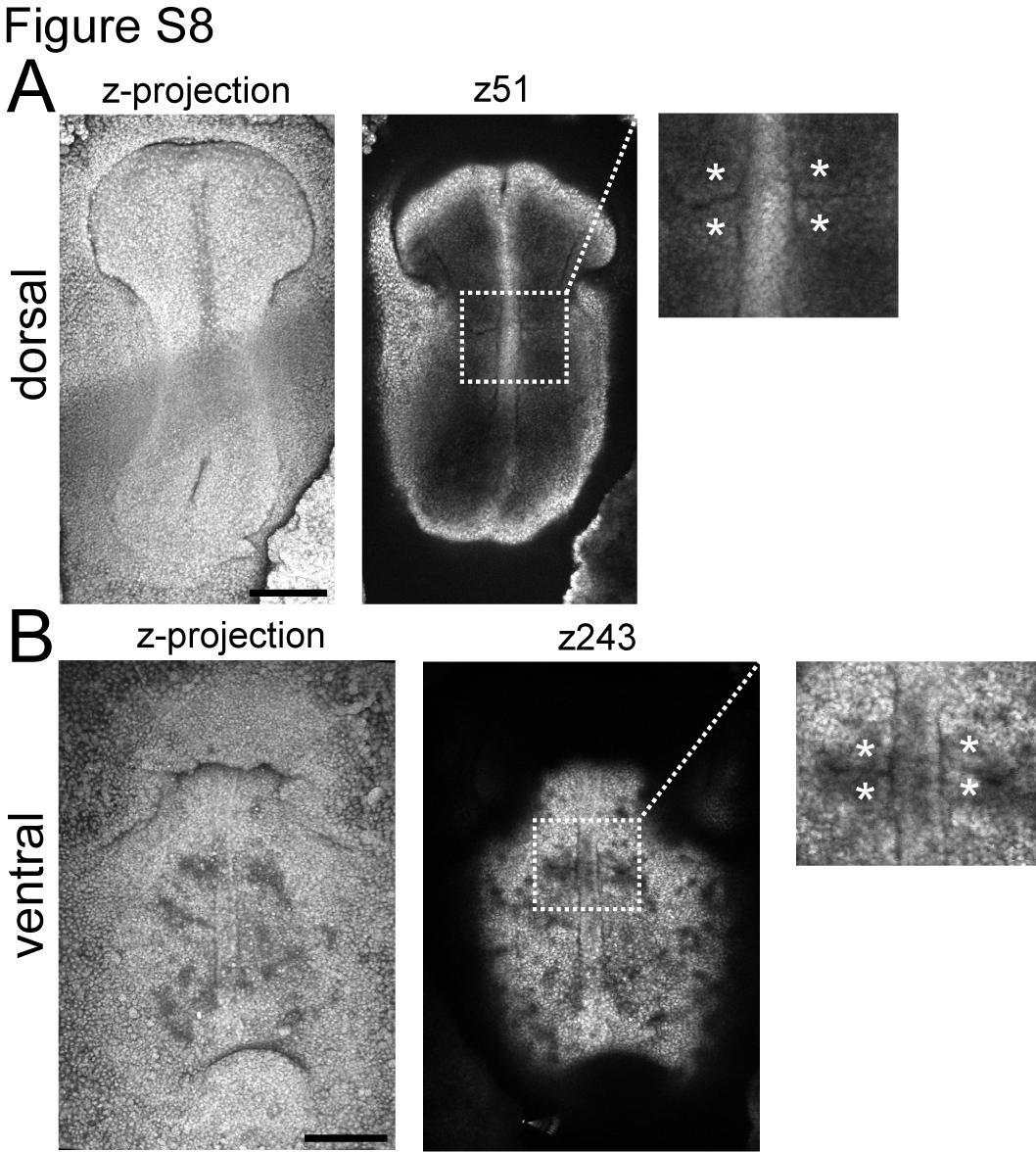

Supplement: Supplementary file 8 — FIGURE S8: Somite formation. DAPI stainings of M149B. (A) M149B dorsal view. Left maximum intensity projection. Right: z51, somites boxed, asterisks in zoom‐in demarcate somites. Scale bar: 250 μm. (B) M149B ventral view. Left maximum intensity projection. Right z243, somites boxed. Asterisks in zoom‐in demarcate somites. Scale bar: 250 μm. [file DVDY-255-145-s012.tif]

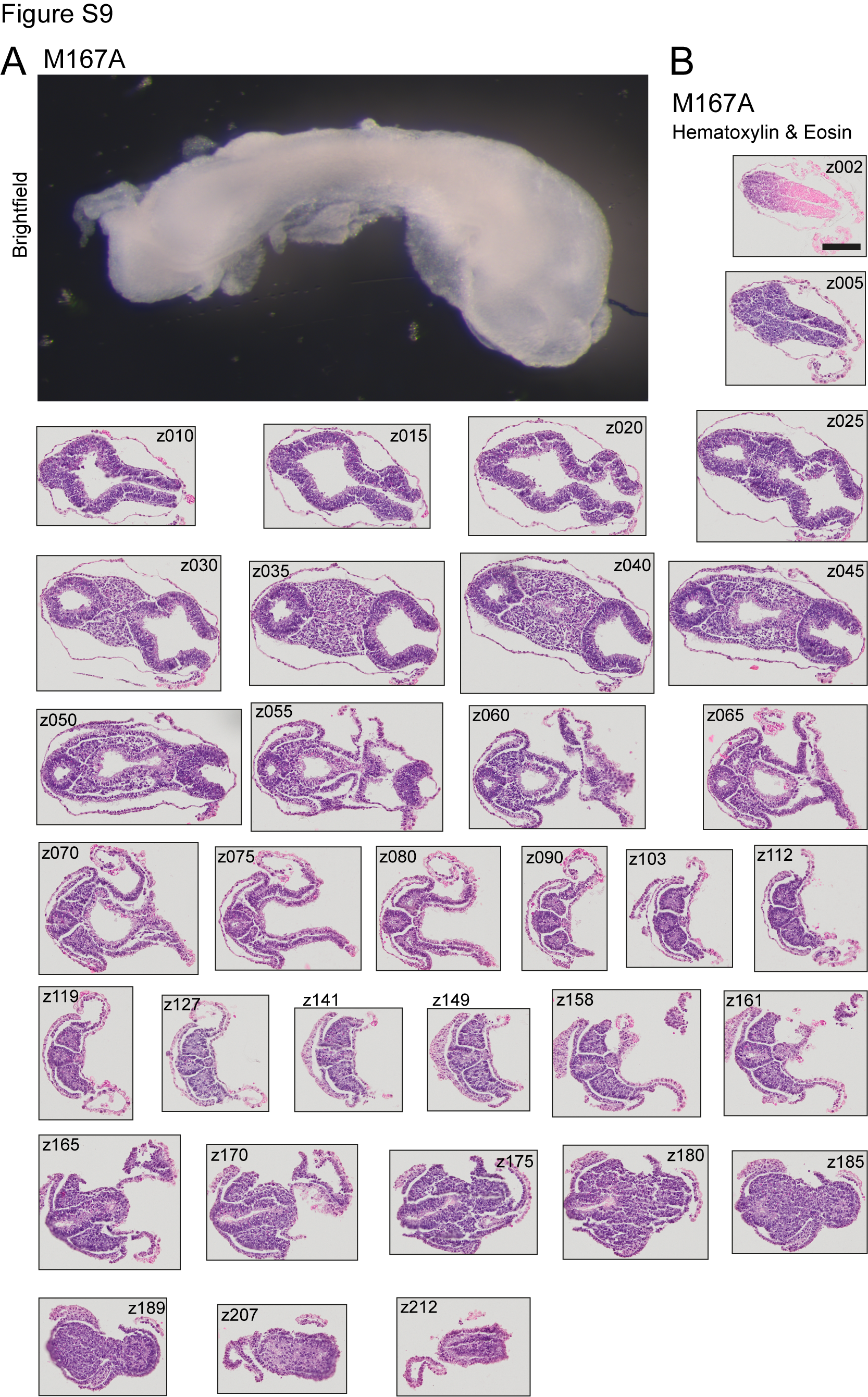

Supplement: Supplementary file 9 — FIGURE S9: Cross sections of neurulation embryo M167A. (A) Brightfield image of dorsal‐right view of M167A. No scale bar. (B) Hematoxylin and eosin staining of paraffin cross sections of M167A. Cross section number is annotated in figure (z002–z212). Scale bar: 100 μm. All imaged have the same scale bar. [file DVDY-255-145-s013.tif]

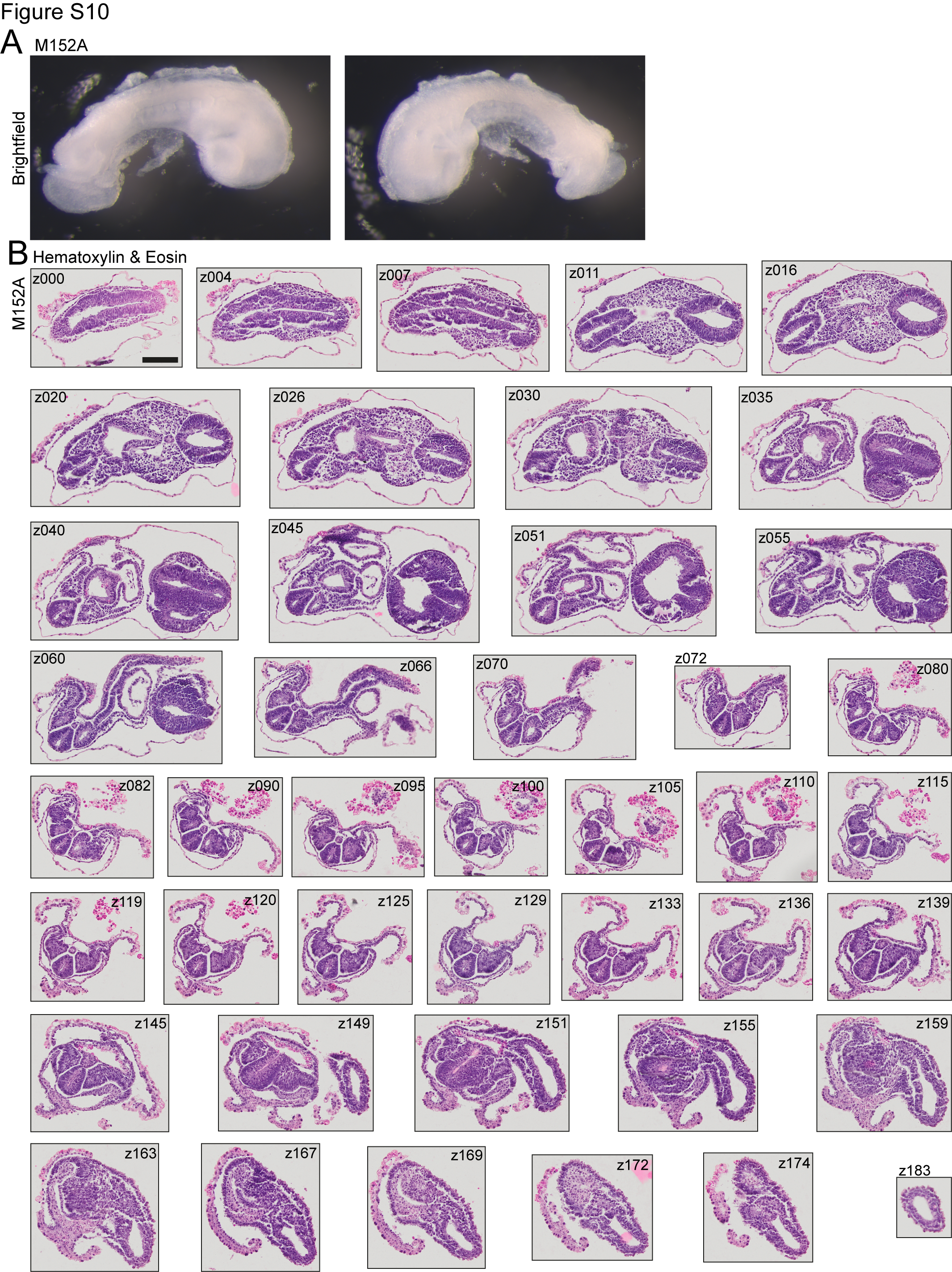

Supplement: Supplementary file 10 — FIGURE S10: Cross sections of late neurulation embryo M152A. (A) Brightfield image of right and left side of M152A. No scale bar. (B) Hematoxylin and eosin staining of paraffin cross sections of M152A. Cross section number is annotated in figure (z000–z183). Scale bar: 100 μm. All imaged have the same scale bar. [file DVDY-255-145-s006.tif]

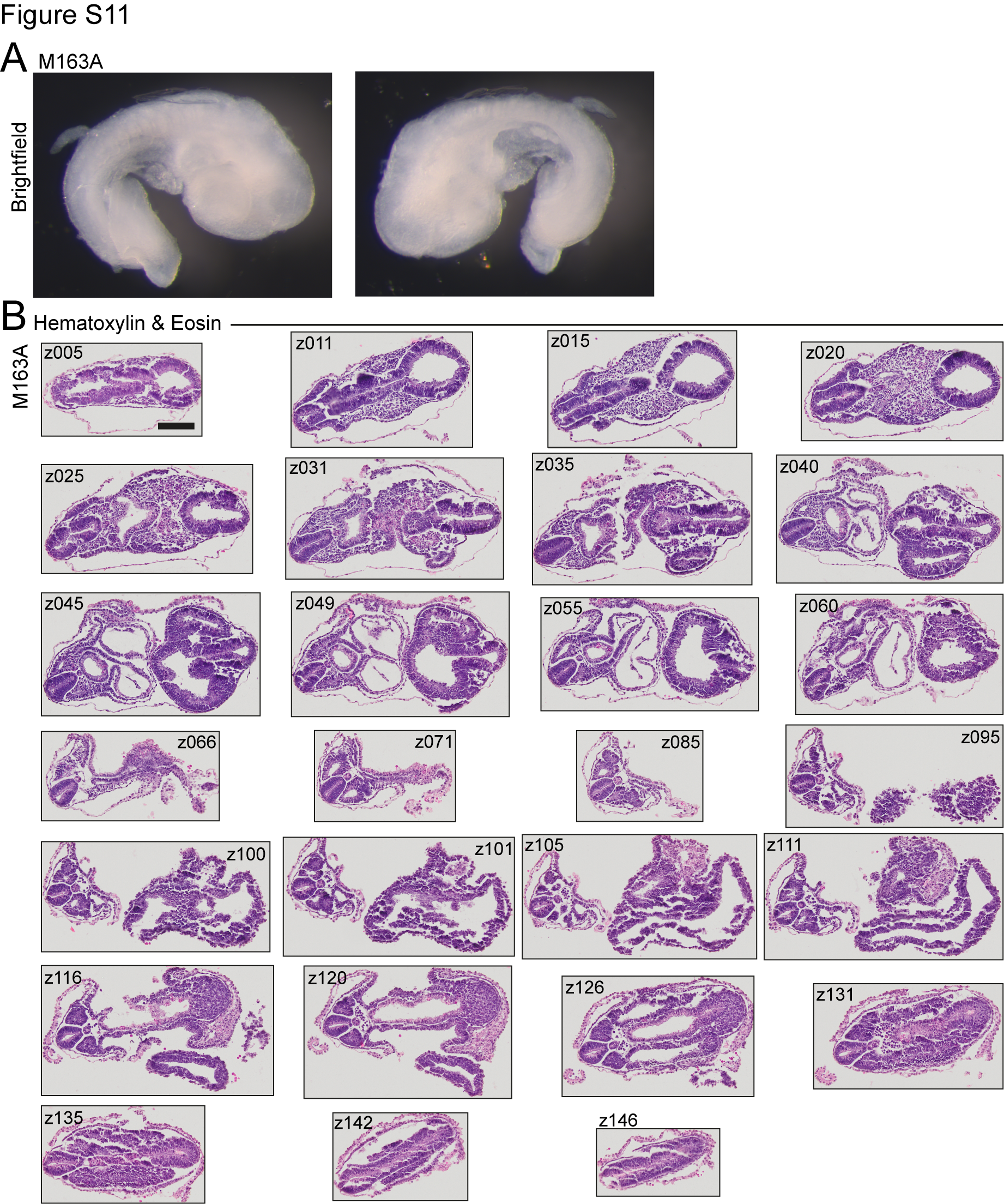

Supplement: Supplementary file 11 — FIGURE S11: Cross sections of late neurulation embryo M163A. (A) Brightfield images of right and left side of M163A. No scale bar. (B) Hematoxylin and eosin staining of paraffin cross sections of M163A. Cross section number is annotated in figure (z005–z146). Scale bar: 100 μm. All imaged have the same scale bar. [file DVDY-255-145-s001.tif]

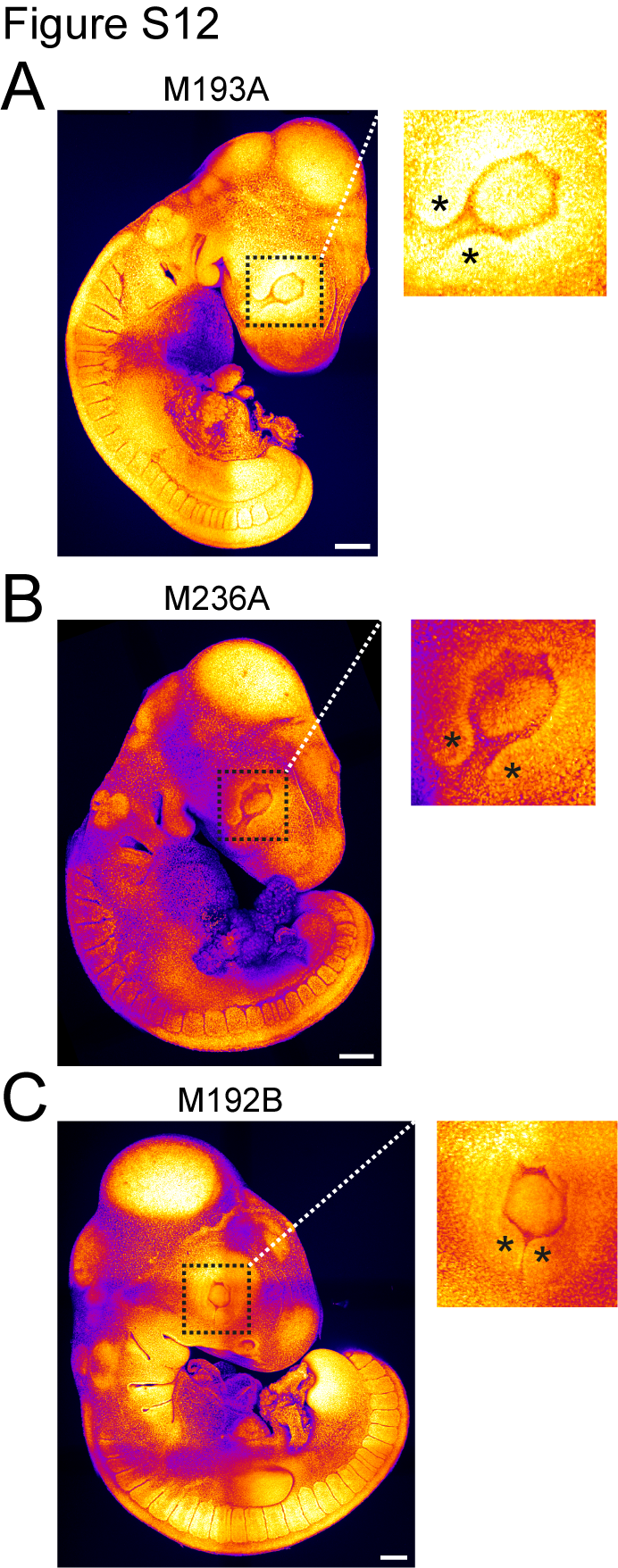

Supplement: Supplementary file 12 — FIGURE S12: Closure of the optic fissure. (A)–(C) Maximum intensity projections of DAPI staining of embryos (A) M193A, (B) M236A, and (C) M192B. Boxes around optical fissure. Asterisks in zoom ins (right column) demarcate the edges of the optical fissure. Scale bar: 200 μm. [file DVDY-255-145-s010.tif]

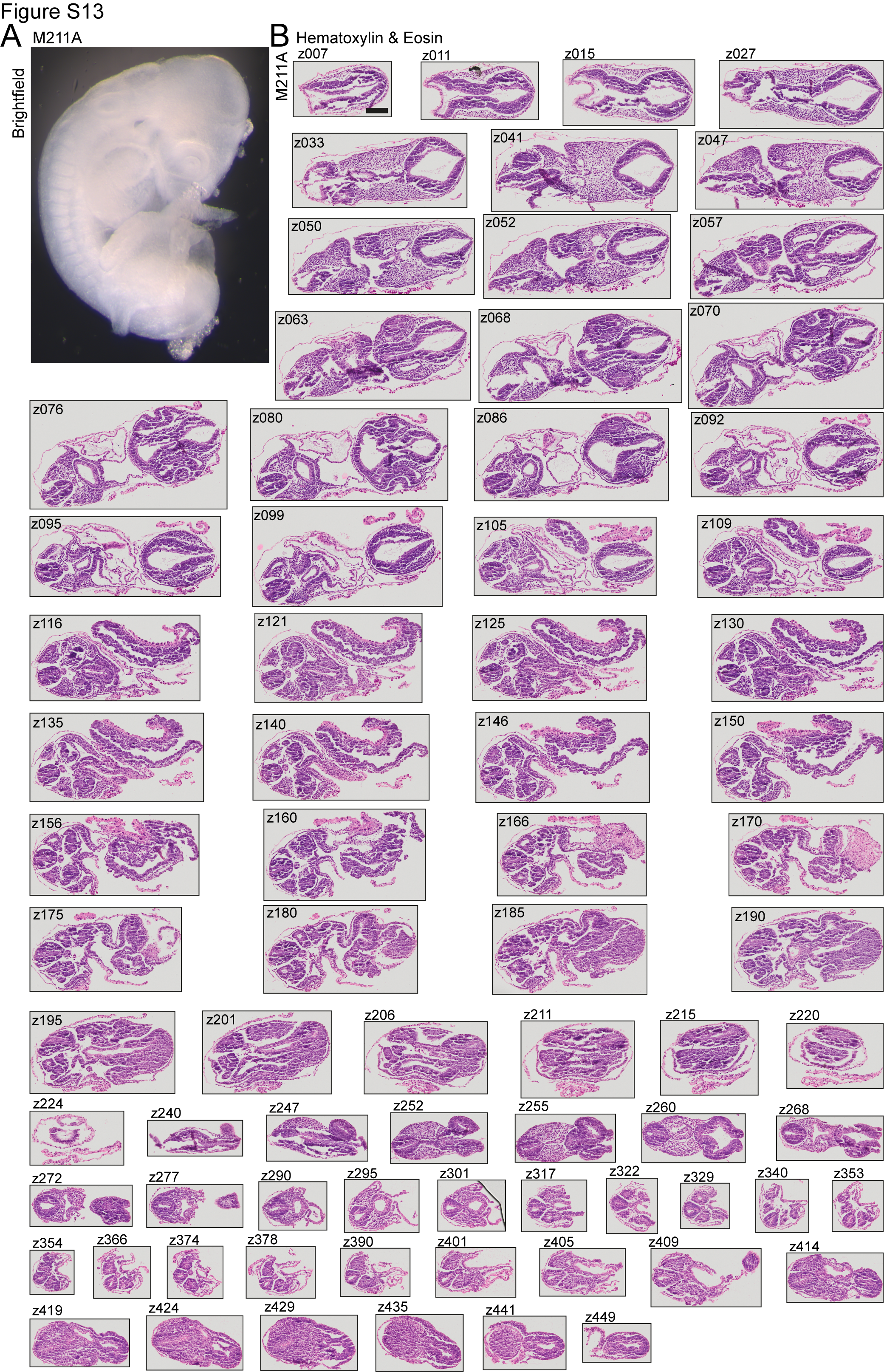

Supplement: Supplementary file 13 — FIGURE S13: Cross sections of organogenesis embryo M211A. (A) Brightfield images of right and left side of M211A. No scale bar. (B) Hematoxylin and eosin staining of paraffin cross sections of M211A. Cross section number is annotated in figure (z007–z449). Scale bar: 100 μm. All imaged have the same scale bar. [file DVDY-255-145-s003.tif]

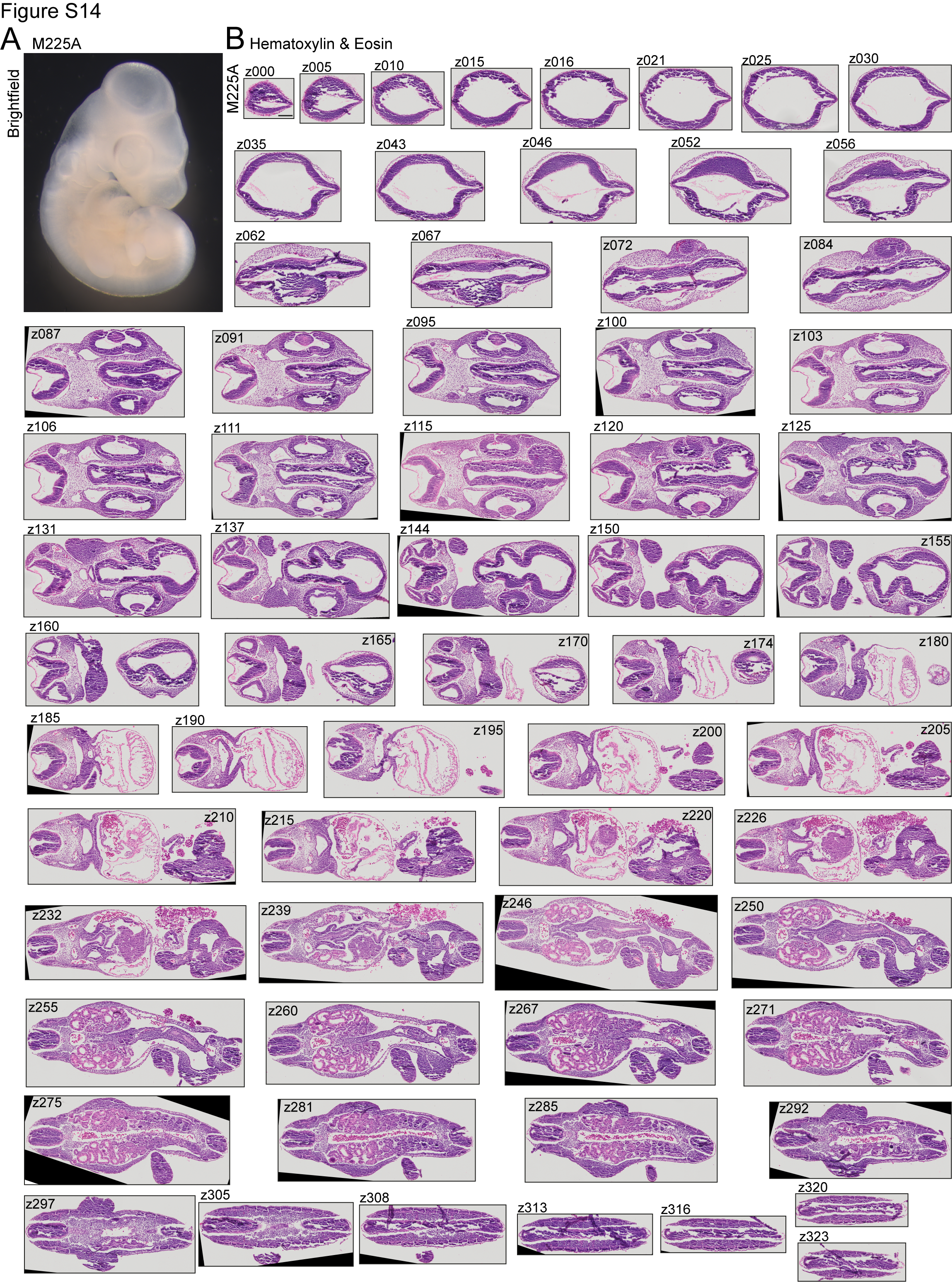

Supplement: Supplementary file 14 — FIGURE S14: Cross sections of organogenesis embryo 225A. (A) Brightfield images of right and left side of M225A. No scale bar. (B) Hematoxylin and eosin staining of paraffin cross sections of M225A. Cross section number is annotated in figure (z000–z323). Scale bar: 100 μm. All imaged have the same scale bar. [file DVDY-255-145-s005.tif]
